# Mouse-adapted SARS-CoV-2 replicates efficiently in the upper and lower respiratory tract of BALB/c and C57BL/6J mice

**DOI:** 10.1007/s13238-020-00767-x

**Published:** 2020-08-04

**Authors:** Jinliang Wang, Lei Shuai, Chong Wang, Renqiang Liu, Xijun He, Xianfeng Zhang, Ziruo Sun, Dan Shan, Jinying Ge, Xijun Wang, Ronghong Hua, Gongxun Zhong, Zhiyuan Wen, Zhigao Bu

**Affiliations:** 1 State Key Laboratory of Veterinary Biotechnology, Harbin Veterinary Research Institute, Chinese Academy of Agricultural Sciences, Harbin, 150069 China; 2 National High Containment Laboratory for Animal Diseases Control and Prevention, Harbin, 150069 China

**Dear Editor,**

As of June, 2020, more than ten million cases of COVID-19 have been reported worldwide. The causative pathogen of the disease is a novel coronavirus named severe acute respiratory syndrome coronavirus 2 (SARS-CoV-2) (World Health Organization, 2020). Animal infection models are important to characterize the infection, pathogenesis, and immunology of SARS-CoV-2, as well as for the development of medications and vaccines against COVID-19. Mice are particularly attractive animal models for their identical genetic background, reliable reproducibility, well characterized biology, and the huge availability of research reagents and knockout animals. Models in inbreed mice such as BALB/c and C57BL/6J (C57), which are widely used in research, are highly desired. The ideal model should mimic the infection of SARS-CoV-2 in humans, in whom the virus efficiently replicates in both the upper and lower respiratory tracts. Several SARS-CoV-2 mouse infection models, including human angiotensin-converting enzyme 2 (ACE2) transgenic mouse models (Bao et al., 2020; Jiang et al., 2020), a BALB/c mouse-adapted virus model (Gu et al., 2020), a reverse genetically modified SARS-CoV-2 infection model (Dinnon III et al., 2020), and recombinant adenovirus-mediated transient expression of human ACE2 mouse models (Hassan et al., 2020; Sun et al., 2020), have been reported that viruses efficiently replicated in the lung. However, none of these reported models showed significant and stable infection in the upper respiratory tract. SARS-CoV-2 infection of the upper respiratory tract in humans is highly associated with the initial infection, the shedding of the offspring virus, and the transmission capability of the disease. Prevention of virus replication in the upper and lower respiratory tract is, therefore, highly desirable and an important aspect of the development of antiviral medications and vaccines against COVID-19. The mouse-adapted virus infected mouse model established here resembling the infection of SARS-CoV-2 in humans will be helpful to achieve the goals.

Firstly, we found SARS-CoV-2/HRB26/human/2020/CHN (HRB26) is able to establish infection in the respiratory tract of BALB/c mice (Fig. S1). 4–6-week-old female BALB/c mice were intranasally (i.n.) infected with HRB26 at a dose of 10^6.2^ plaque forming unit (PFU). On day 3 post inoculation (p.i.), the nasal turbinates and lungs were respectively collected and homogenized for viral RNA detection by qPCR and virus titration in Vero E6 cells. HRB26 only infected the nasal turbinates of 2 of the 3 inoculated mice and the lung of 1 of the 3 inoculated mice (Fig. S1). We serially passaged HRB26 in 4–6-week-old female BALB/c mice. A mixture of nasal turbinate and lung homogenate from the mouse of each passage with the highest viral RNA copies was used to inoculate three mice via intranasal inoculation. The viral RNA loads increased by passages in the nasal turbinates (Fig. S1A) and lungs (Fig. S1B). The infectious titres in the nasal turbinates and lungs at passage 14 (P14) were 10^5^ PFU/g and 10^6.7^ PFU/g on day 3 p.i., respectively (Fig. S1C and S1D). The virus of P14 was propagated in Vero E6 cells and the resultant mouse-adapted virus was designated as HRB26M (10^5.7^ PFU/mL). The 50% mouse infectious dose (MID_50_) of HRB26M in 4–6-week-old female BALB/c mice was 1.4 PFU (Fig. S2). In the mice infected i.n. with HRB26M, the viral RNA was detected in the nasal turbinates on day 3, 5, and 7 p.i., and the infectious virus was detected on day 3 and 5 p.i. (Fig. 1A and 1B). The viral RNA was also detected in the heart, liver, kidney and spleen on day 3 p.i., but not on day 5 and day 7 p.i., respectively (Fig. S3A and S3B).

**Figure 1:**
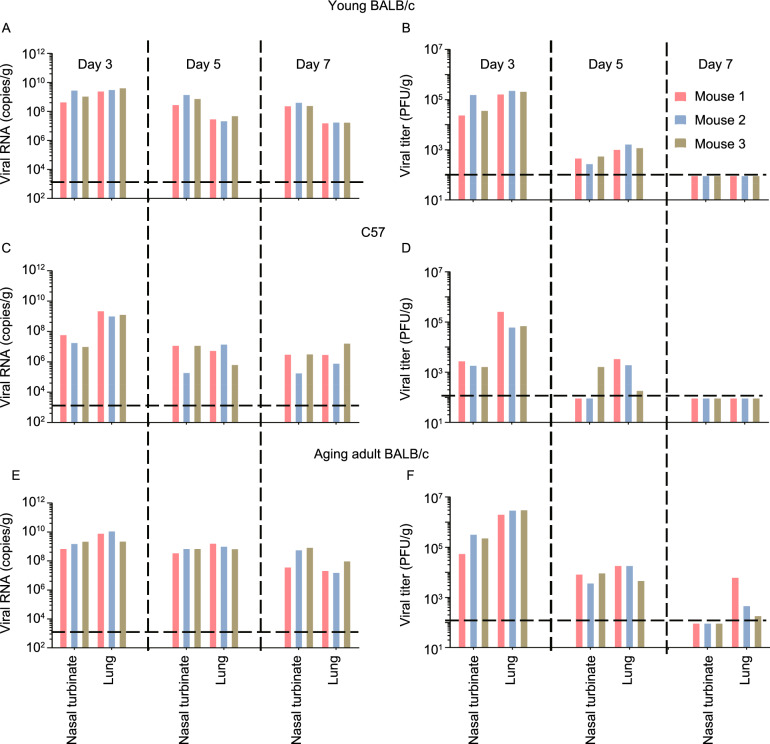

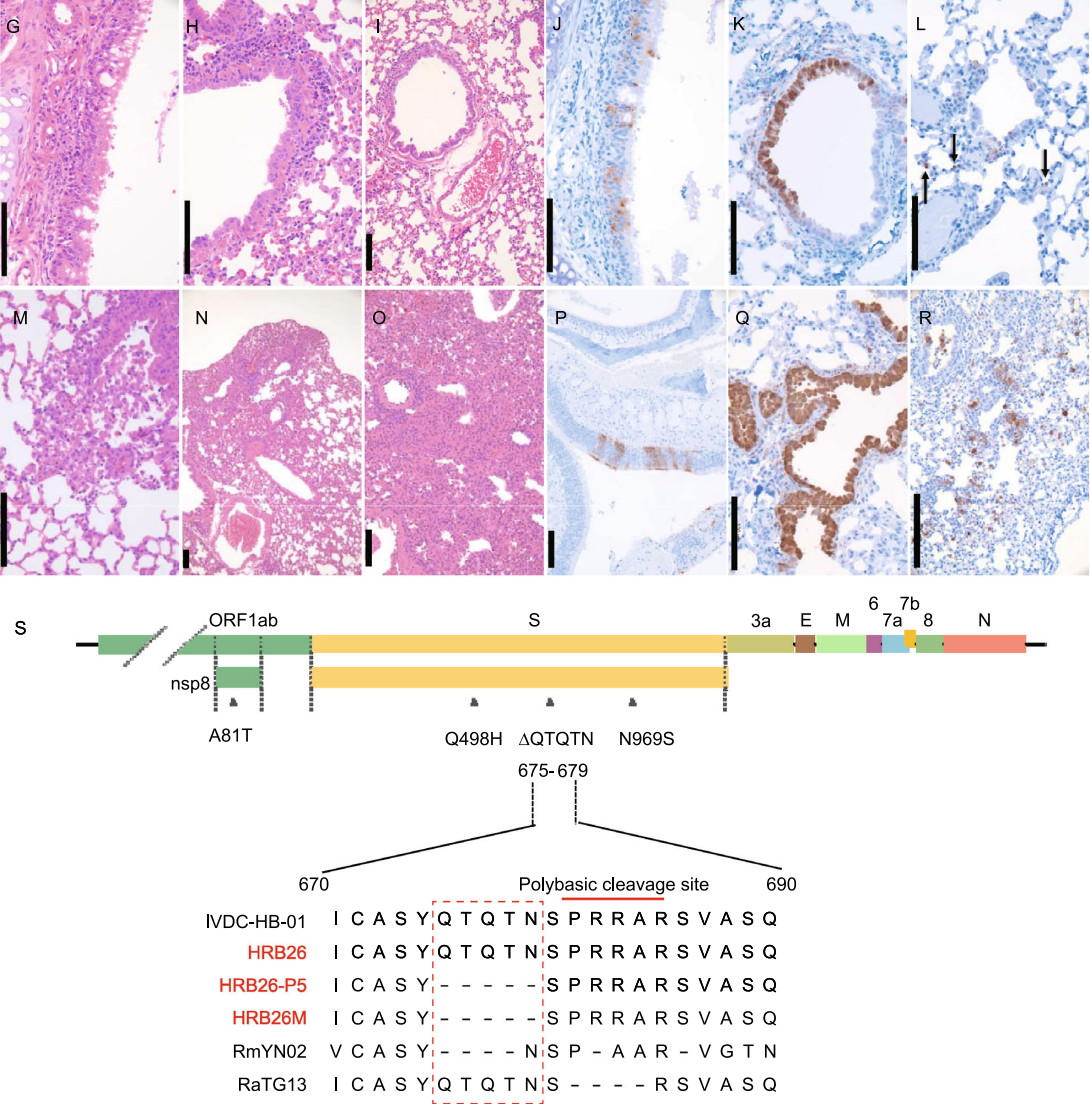
**Characterization of Mouse-adapted SARS-CoV-2 HRB26M in mice**. Groups of nine 4–6-week-old female BALB/c mice (A, B), 4–6-week-old female C57 mice (C, D) or 8–9-week-old male BALB/c mice (E, F) were inoculated i.n. with 10^4.4^ PFU of HRB26M in a volume of 50 μL. On days 3, 5, and 7 p.i., three mice were each euthanized, and their nasal turbinates and lungs were collected for virus detection. The viral RNA copies (A, C, E) and infectious titres (B, D, F) in each organ were detected by qPCR and virus titration. The horizontal dashed lines indicate the limit of detection. Histopathologic and immunohistochemical studies were performed on samples from the HRB26M-inoculated young female mice (G–L) and aging adult male mice (M–R). The nasal respiratory mucosa epithelium exhibited an abnormal arrangement with loss of cilia accompanied by monocyte and lymphocyte infiltration in the lamina propria on day 3 p.i. (G). Diffuse degeneration of the epithelial cells of the bronchiole and moderate peripheral inflammatory cell infiltration were observed on day 5 p.i. (H). Congestion in the interalveolar septa and perivascular edema were commonly observed in the lungs on day 3 p.i. (I). Viral antigen was detected in the epithelium of the nasal respiratory mucosa (J), the epithelial cells of the bronchiole (K), and the alveolar septa cells (L) on day 3 p.i.. Degeneration and necrosis of the epithelial cells of the bronchiole and alveolar duct, and monocyte and lymphocyte infiltration in the lumen of the lungs were observed on day 3 p.i. (M). Perivascular edema and inflammation (N) and monocyte and lymphocyte congestion in the interalveolar septa and alveolar lumen (O) of the lung were observed on day 5 p.i. Viral antigens were detected in the epithelium of the nasal mucosa (P), bronchiole (Q) and alveolar septa (R) on day 3 p.i. Bars, 100 μm. (S) Location of the mutations and deletion in the genome of HRB26M and the alignment of the sequence near the polybasic cleavage site of the S protein of different SARS-CoV-2 strains. IVDC-HB-1, BetaCoV/Wuhan/IVDC-HB-01/2019|EPI_ISL_402119; RmYN02, BetaCoV/Rm/Yunnan/YN02/2019|EPI_ISL_412977; RaTG13, BetaCoV/bat/Yunnan/RaTG13/2013|EPI_ISL_402131

Mild pathological changes were observed in the respiratory tract of 4–6-week-old BALB/c mice infected i.n. with HRB26M (Fig. 1G–I). Viral antigens were detected in the epithelium of the nasal respiratory mucosa (Fig. 1J), the epithelial cells of the bronchiole (Fig. 1K) and the alveolar septa cells (Fig. 1L) on day 3 p.i. These results demonstrate that SARS-CoV-2 successfully adapted and efficiently infected the upper and lower respiratory tract of young BALB/c mice.

SARS-CoV-2 infection causes more serious disease and higher mortality in people older than 65 years (Guan et al., 2020). We then assessed the infectivity and pathogenicity of HRB26M in 8–9-month-old (aging adult) male BALB/c mice. Aging adult mice inoculated i.n. with 10^4.4^ PFU of HRB26M showed transient weight loss on days 2 and 3 p.i. and recovered thereafter, similar to young mice (Fig. S4A and S4B). The viral RNA loads in the nasal turbinates were 10^8.7~9.5^ copies/g, 10^8.5~9.2^, and 10^8.4~8.7^ copies/g on days 3, 5, and 7 p.i., respectively (Fig. 1E). The lungs had peak viral RNA loads of 10^9.5~9.7^ on day 3 p.i., and then slightly decreased loads on days 5 and 7 p.i. (Fig. 1E). Viral RNAs were also detected in the heart, brain, kidney, spleen, small intestine, and liver in some or all three inoculated mice on days 3 and 5 p.i. (Fig. S3E). Infectious virus titres in the nasal turbinates and lungs peaked on day 3 p.i. The average PFU titres were 3 times higher than those in the nasal turbinates of young female mice, but about 10 times higher in the lungs of the older mice than those of the younger mice (Fig. 1B and 1F). These results indicate that the mouse-adapted virus HRB26M infects aging adult BALB/c mice more extensively than young BALB/c mice.

Histopathologic study revealed that aging adult mice developed moderate-to-severe pathological changes in the lungs after infection (Fig. 1M–O). Compared to younger mice, much stronger viral antigen signals were detected in the epithelium of the nasal mucosa (Fig. 1P), the bronchiole (Fig. 1Q) and the alveolar septa (Fig. 1R) on day 3 p.i., which is coincident with the results of virus loads. The results demonstrate that HRB26M causes more severe pathological changes in the respiratory tract in aging adult mice.

C57 is the most widely used line for generating gene knockout mouse. We next tested the infectivity of HRB26M in C57 mice. Group of six 4–6-week-old C57 mice were inoculated i.n. with 10^4.4^ PFU of HRB26M. The average viral loads in the nasal turbinates were 10^8.2^ copies/g, 10^7.6^ and 10^6.5^ copies/g of viral RNA and 10^3.3^ PFU/g, 10^2.9^ PFU/g and negative detection of infectious virus titres on days 3, 5 and 7 p.i., respectively (Fig. 1C and 1D). The average viral loads in the lungs were 10^7.9^ copies/g, 10^7.0^ and 10^6.9^ copies/g of viral RNA and 10^4.4^ PFU/g, 10^2.3^ PFU/g and negative detection of infectious virus titres on days 3, 5 and 7 p.i., respectively. These findings indicate that HRB26M efficiently infects both the upper and lower respiratory tract of C57 mice.

We next compared the whole genome of mouse passage 5 (P5) and P14 viruses with that of HRB26. Two nonsynonymous mutations, Q498H and N969S, and a deletion of five amino acids (675QTQTN679) that flanks the cleavage site in the *S* gene were found in P5 virus (Fig. 1S). In P14 virus, an additional mutation of A81T in the *nsp8* gene encoding the cofactor of RdRp (Kirchdoerfer and Ward, 2019) happened (Fig. 1S). The position 498 in the receptor binding domain is important for the selective binding of the S protein to the ACE2 receptor (Dinnon III et al., 2020). The Q498H mutation in P5 virus may be a key for the early adaptation. Interestingly, the deletion of residues 675–679 in S has previously been reported in SARS-CoV-2 virus after passage in cell culture and in original human clinical samples (Liu et al., 2020). Notably, a similar deletion of QTQT at positions 675–678 of S has been reported in bat-derived SARS-like strains (Zhou et al., 2020). It will be important to clarify whether this deletion plays a role in the interspecies host transmission and adaption of SARS-CoV-2. The nsp8-A81T mutation, which was found in P14 virus but not in P5 or P10 viruses, may have a role in the enhanced replication of HRB26M. How these mutations and deletion affect mouse adaption remains to be investigated.

Remdesivir is a nucleoside analogue with antiviral activity against SARS-CoV-2 (Wang et al., 2020a). We confirmed that HRB26 and HRB26M have similar sensitivity to remdesivir in Vero E6 cells (Fig. 2A and 2B). Clinical trials have shown that remdesivir is a promising antiviral drug for the treatment of COVID-19 (Beigel et al., 2020; Wang et al., 2020b). However, the efficacy of remdesivir to prevent the replication of SARS-CoV-2 in the upper respiratory tract remains to be investigated. Here, we assessed the antiviral efficacy of remdesivir in HRB26M-infected BALB/c mouse model.

**Figure 2:**
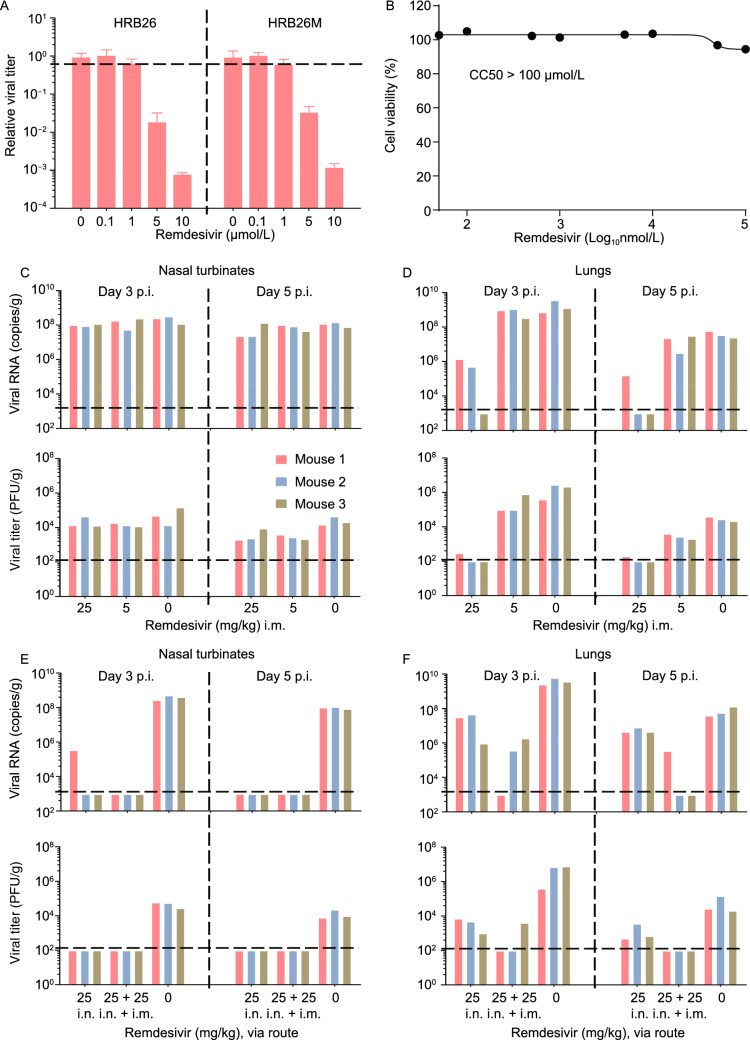
**Evaluation of remdesivir against SARS-CoV-2 infection** ***in vitro*** **and** ***in vivo***. Vero E6 cells were pretreated with the indicated concentrations of remdesivir or DMSO (0 μmol/L) for one hour. The cells were then infected with HRB26 or HRB26M at an MOI of 0.005 and incubated for 1 h at 37 °C. After the cells were washed with PBS, virus growth medium containing the indicated concentrations of remdesivir or DMSO was added. Relative viral titers at 24 h post-inoculation (A) were calculated from the ratios for the mock-treated counterparts. Data shown are the mean values with standard deviations for the results of three independent experiments. The horizontal dashed lines indicate the 50% inhibition of remdesivir against SARS-CoV-2. (B) Cell viability was determined 24 h post-inoculation by using the Cell Titer-Glo kit following the manufacturer’s instructions. Groups of six 4–6-week-old female BALB/c mice were treated i.m. (C and D) with a loading dose of 50 or 10 mg/kg remdesivir, followed by a daily maintenance dose of 25 or 5 mg/kg. Alternatively, groups of six mice were treated with a loading dose of 50 mg/kg remdesivir, followed by a daily maintenance dose of 25 mg/kg i.n. alone or a combination of i.n. and i.m. (E and F). Control mice were administrated vehicle solution (12% sulfobutylether-β-cyclodextrin, pH 3.5) daily, in parallel (0 mg/kg). One hour after administration of the loading dose of remdesivir or vehicle solution, the mice were inoculated i.n. with 10^3.6^ PFU of HRB26M in a volume of 50 μL. On days 3 and 5 p.i., three mice in each group were euthanized and their nasal turbinates and lungs were collected. The viral RNA copies and infectious titres in the nasal turbinates (C, E) and lungs (D, F) were detected by qPCR and virus titration. The concentrations of the daily maintenance doses are shown. The horizontal dashed lines indicate the limit of detection

Groups of six 4–6-week-old female BALB/c mice were intramuscularly (i.m.) administered remdesivir at a dose of 25 mg/kg or 5 mg/kg. For the mock-treatment control, mice were administrated vehicle solution (12% sulfobutylether-β-cyclodextrin, pH 3.5) daily. One hour after remdesivir administration, mice were inoculated i.n. with 10^3.6^ PFU of HRB26M. In the high-dose remdesivir-treated group, the viral RNA was detected in the lungs of 2 of the 3 mice on day 3 p.i. and 1 of the 3 mice on day 5 p.i.; however, the viral RNA loads and the virus titers were at least 1,000-times lower than those in the mock-treated mice (Fig. 2C and 2D). In the low-dose remdesivir-treated group, the virus yields were only slightly reduced when compared to those in mock-treated mice. However, virus replication in the nasal turbinates was not significantly affected after remdesivir treatment in both the high and low dose groups (Fig. 2C and 2D). These data indicate that remdesivir treatment via intramuscular route efficiently inhibits the replication of SARS-CoV-2 in the lungs in a dose-dependent manner, but not in the nasal turbinates of BALB/c mice.

We then tested whether intranasal administration could improve the efficacy of remdesivir to inhibit the replication of SARS-CoV-2 in the upper respiratory tract. Mice were treated i.n. with remdesivir at a dose of 25 mg/kg and the virus replication in the nasal turbinates was completely blocked. No infectious virus was detected on days 3 and 5 p.i., although viral RNA was detected in 1 of the 3 mice on day 3 p.i. (Fig. 2E). Compared to the vehicle-treated group, intranasal administration of remdesivir also significantly inhibited virus replication in the lungs (*P* < 0.01) (Fig. 2F). Moreover, remdesivir treatment via the combined intranasal and intramuscular routes completely prevented virus replication in the nasal turbinates and significantly inhibited virus replication in the lungs (*P* < 0.01). Infectious virus was recovered from the lungs of 1 of the 3 mice on day 3 p.i., but not from any of mice on day 5 p.i., although viral RNAs were detected from the lungs of 2 of the 3 mice on day 3 p.i. and 1 of the 3 mice on day 5 p.i. (Fig. 2F). The data suggested that intranasal administration of remdesivir successfully prevents the replication of virus in the upper respiratory tract. Combined intranasal and intramuscular treatment efficiently inhibits replication of virus in the upper and lower respiratory tract of mice. Our results indicated the difference in the pharmacokinetics of remdesivir between the upper and lower respiratory tract. Combined intramuscular and intranasal administration of remdesivir should be developed and considered for future clinic practice.

In conclusion, we generated a mouse-adapted SARS-CoV-2 resembling the infection of SARS-CoV-2 in humans. The mouse-adapted virus replicates efficiently in the upper and lower respiratory tract in BALB/c and C57BL/6J mice. The mouse-adapted virus infection model will be a powerful tool for research and development against COVID-19.

## Electronic supplementary material

Below is the link to the electronic supplementary material.

Supplementary material 1 (PDF 844 kb)
