# Supplementary material for: Mouse-adapted SARS-CoV-2 replicates efficiently in the upper and lower respiratory tract of BALB/c and C57BL/6J mice
Source: Protein Cell. 2020 Aug 4;11(10):776–82. doi: 10.1007/s13238-020-00767-x (PMC7401472; doi:10.1007/s13238-020-00767-x)
Supplement: 13238_2020_767_MOESM1_ESM — SUPPLEMENTARY MATERIALS [file 13238_2020_767_moesm1_esm.pdf]

SUPPLEMENTAL MATERIALS

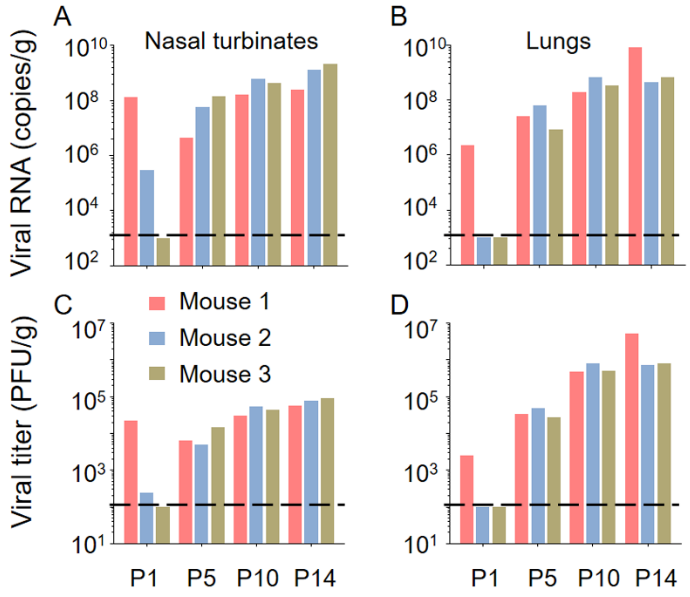

Figure S1 Adaptation of SARS-CoV-2 in BALB/c mice. 4–6-week-old female mice were inoculated i.n. with $10^{6.2}$ PFU of SARS-CoV-2 virus HRB26. On day 3 p.i., nasal turbinates and lungs were collected and a mixture of nasal turbinate and lung homogenates with the highest viral RNA copies was defined as P1 and serially passaged to 3 mice by intranasal inoculation until P14. Viral RNA copies (A, B) and infectious titres (C, D) in the nasal turbinates (A, C) and lungs (B, D) from the indicated passages were detected by qPCR and virus titration. The horizontal dashed lines indicate the limit of detection.

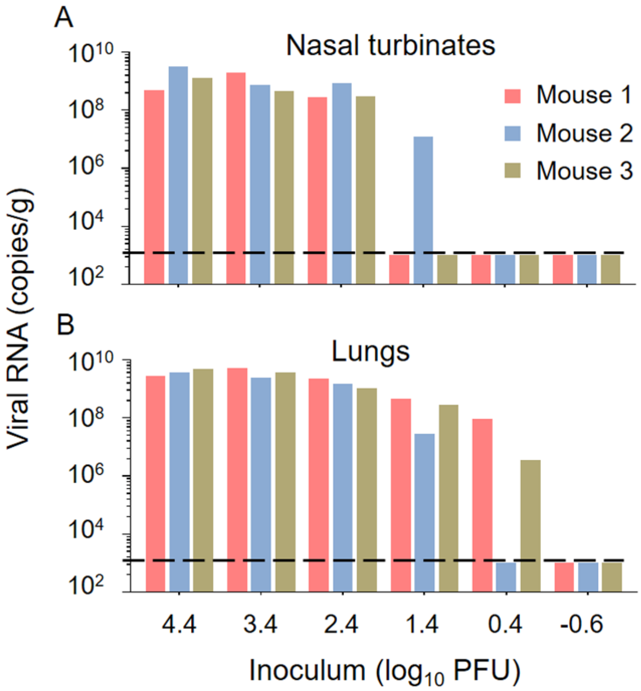

Figure S2 50% mouse infectious dose (MID_{50}) of mouse-adapted virus in young female BALB/c mice. Groups of three 4–6-week-old female BALB/c mice were inoculated i.n. with 10-fold serially diluted HRB26M. On day 3 p.i., three mice in each group were euthanized and the viral RNA copies in the nasal turbinates (A) and lungs (B) were detected by qPCR. The horizontal dashed lines indicate the limit of detection.

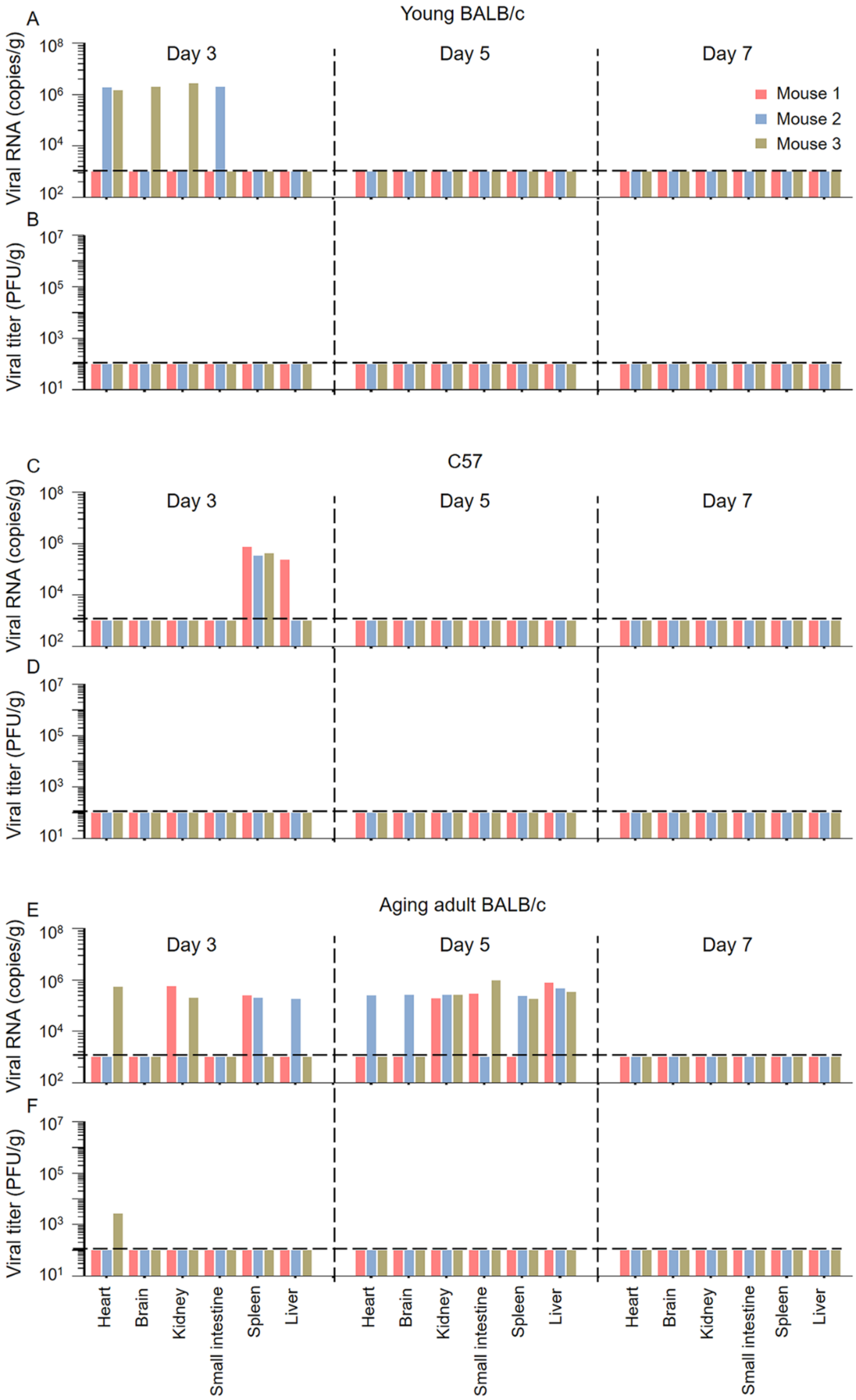

A
Young BALB/c
Viral RNA (copies/g)
Day 3
Day 5
Day 7
Mouse 1
Mouse 2
Mouse 3
B
Viral titer (PFU/g)
C
C57
D
E
Aging adult BALB/c
F
Heart
Brain
Kidney
Small intestine
Spleen
Liver

Figure S3 Replication of mouse-adapted SARS-CoV-2 virus in the organs of young and aging adult male BALB/c and C57 mice. Groups of nine 4–6-week-old female BALB/c (A, B), 4–6-week-old female C57 (C, D) or 8–9-month-old male BALB/c mice (E, F) were inoculated i.n. with $10^{4.4}$ PFU of HRB26M in a volume of 50 μL. On days 3, 5, and 7 p.i., three mice in each group were euthanized. The viral RNA copies (A, C, E) and infectious titres (B, D, F) in the hearts, brains, kidneys, small intestines, spleens, and livers were detected by qPCR and virus titration. The horizontal dashed lines indicate the limit of detection.

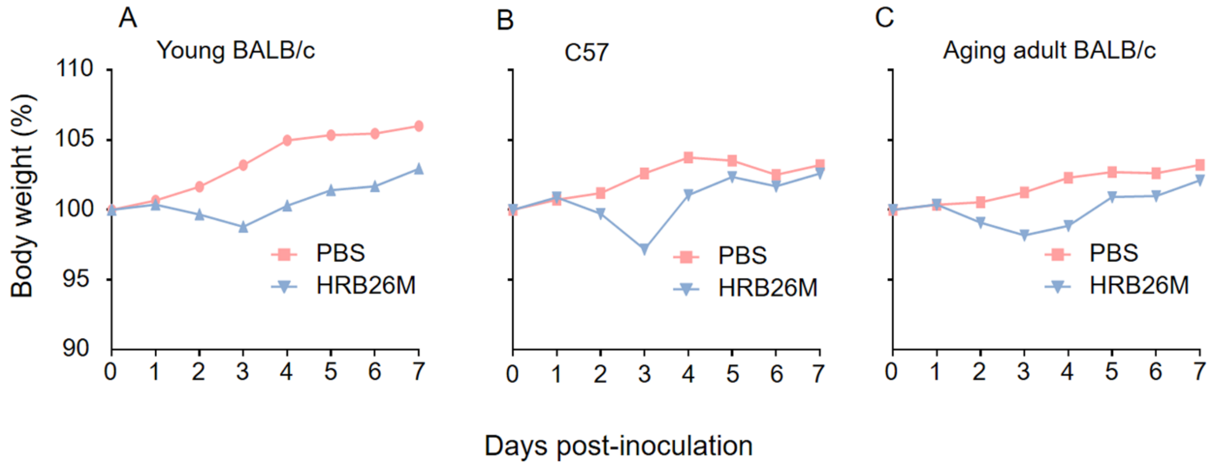

Figure S4 Body weight changes. Groups of three 4–6-week-old (young) female BALB/c (A), 4–6-week-old female C57 (B), or 8–9-month-old (aging adult) male BALB/c (C) mice were lightly anesthetized with CO_2 and i.n. inoculated with $10^{4.4}$ PFU of HRB26M or PBS. Body weights were monitored daily for 7 days and are presented as a percentage of the weight on the day of inoculation (day 0).

MATERIALS AND METHODS

Biosafety and Facility

All experiments with infectious SARS-CoV-2 were performed in the biosafety level 4 and animal biosafety level 4 facilities in the Harbin Veterinary Research Institute (HVRI) of the Chinese Academy of Agricultural Sciences (CAAS), which is approved for such use by the Ministry of Agriculture and Rural Affairs of China.

Cells and Viruses

Vero E6 cells were maintained in Dulbecco's modified Eagle's medium (DMEM) containing 10% fetal bovine serum (FBS) and antibiotics and incubated at 37°C with 5% CO_2. SARS-CoV-2/HRB26/human/2020/CHN (HRB26, GISAID access no. EPI_ISL_459909) was isolated from a patient in Vero E6 cells. Viral stocks were prepared in Vero E6 cells with DMEM containing 5% FBS. Mouse-adapted SARS-CoV-2/HRB26/human/2020/CHN (HRB26M, GISAID access no. EPI_ISL_459910) was obtained by serially passaging the HRB26 virus in 4–6-week-old female mice until passage 14 and was propagated in Vero E6 cells. Infectious virus titers were determined by using a plaque forming unit (PFU) assay in Vero E6 cells.

qPCR and sequencing of viral genomes

Viral genomic RNA of SARS-CoV-2 was extracted by using a QIAamp vRNA Minikit (Qiagen, Hilden, Germany). Reverse transcription was performed by using the HiScript II Q RT SuperMix (Vazyme, Nanjing, China) for qPCR. qPCR was performed to quantitate the viral N gene RNA copies by using the Applied Biosystems QuantStudio 5 Real-Time PCR System (Thermo Fisher Scientific, Waltham, MA, USA) with Premix Ex Taq (probe qPCR)

(Takara, Dalian, China). The *N* gene-specific primers (forward, 5'-GGGGAACTTCTCCTGCTAGAAT-3'; reverse, 5'-CAGACATTTTGCTCTCAAGCTG-3') and probe (5'-FAM-TTGCTGCTGCTTGACAGATT-TAMRA-3') were utilized according to the information provided by the National Institute for Viral Disease Control and Prevention, China (http://nmdc.cn/nCoV). The amount of vRNA for the target SARS-CoV-2 *N* gene was normalized to the standard curve obtained by using a plasmid (pBluescriptIISK-N, 4,221 bp) containing the full-length cDNA of the SARS-CoV-2 *N* gene. Viral genome sequencing was performed on an Applied Biosystems DNA analyzer. Sequences were assembled and analyzed with the Seqman module of the DNAStar package.

Mouse study

The mice for this study, 4–6-week-old female and 8–9-month-old male BALB/c, and 4–6-week-old female C57BL/6J (C57) were obtained from Beijing Charles River Labs (Beijing, China). Mice were lightly anesthetized with CO_2 and intranasally (i.n.) inoculated with 50 μL dilutions of SARS-CoV-2. Body weights and clinical symptoms were monitored daily. On days 3, 5, or 7 post-inoculation (p.i.), animals were euthanized and their organs, including nasal turbinates, lungs, hearts, brains, kidneys, small intestines, spleens, and livers, were collected for viral RNA detection by qPCR, virus titration by use of a PFU assay, and histopathological study.

To determine the 50% mouse infectious dose (MID_{50}) of HRB26M virus, groups of three young female mice were inoculated i.n. with 10-fold serially diluted HRB26M virus. On day 3 p.i., three mice in each group were euthanized and their nasal turbinates and lungs were collected for virus detection; 50% mouse infectious dose (MID50) at day 3 p.i. were

calculated by the method of Reed and Muench (Reed and Muench, 1938).

Histopathologic and immunohistochemical studies

Animal tissues were fixed in 10% neutral-buffered formalin, embedded in paraffin, and cut into 4-μm sections. The sections were stained with hematoxylin-eosin for histopathologic observation. The sections used for immunohistochemistry were dewaxed in xylene and hydrated through a series of descending concentrations of alcohol to water. For viral antigen retrieval, sections were immersed in citric acid/sodium citrate solution, treated with 3% hydrogen peroxide, and blocked with 8% skim milk. Primary rabbit anti-SARS-CoV-2 nucleoprotein monoclonal antibody (1:500; Frdbio, Wuhan, China) and HRP-conjugated anti-rabbit IgG (whole molecule) secondary antibody (1:600, Sigma-Aldrich) were used. Immunostaining was visualized with DAB and counterstained with hematoxylin.

Evaluation of antiviral activity in cells

Remdesivir (GS-5734) was obtained from Hanxiang BioTec (Shanghai, China). Vero E6 cells were pretreated with the indicated concentrations of remdesivir in DMSO or with DMSO alone for 1 hour. The cells were then infected with HRB26 or HRB26M at an MOI of 0.005 and incubated for 1 hour at 37°C. Cells were washed with PBS and virus growth medium containing the indicated amounts of remdesivir in DMSO or DMSO alone was added. Supernatants were collected at 24 hours post-infection for virus titration by use of a PFU assay in Vero E6 cells. Relative viral titers were calculated on the basis of the ratios to the viral titers in the mock-treated counterparts. Data shown are the mean values with standard deviations for the results of three independent experiments.

Cell viability was determined by using the Cell Titer-Glo kit (Promega, Madison, WI,

USA) following the manufacturer's instructions. Briefly, Vero E6 cells were seeded in 96-well plates with opaque walls. After 12 to 16 hours, the indicated concentrations of remdesivir in DMSO were added. Twenty-four hours later, Cell Titer-Glo reagent was added to each well. Luminescence was measured with a GloMax 96 Microplate Luminometer (Promega, Madison, WI, USA).

Evaluation of antiviral activity in mice

Groups of six 4–6-week-old female mice were treated intramuscularly (i.m.) with a loading dose of 50 mg/kg（high dose） or 10 (low dose) mg/kg remdesivir, followed by a daily maintenance dose of 25 mg/kg（high dose） or 5 mg/kg (low dose). Alternatively, mice were treated i.n. alone or a combination of i.n. and i.m. with a loading dose of 50 mg/kg remdesivir, followed by a daily maintenance dose of 25 mg/kg. As a control, mice were administered vehicle solution (12% sulfobutylether-β-cyclodextrin, pH 3.5) daily. One hour after administration of the loading dose of remdesivir or vehicle solution, each mouse was inoculated i.n. with$10^{3.6}$ PFU of HRB26M in 50 μL. Three mice from each group were euthanized on days 3 and 5 p.i.. The nasal turbinates and lungs were collected for virus detection by qPCR and PFU assay.
